# Supplementary material for: Bioinformatics and biomedical informatics with ChatGPT: Year one review
Source: Quant Biol. 2024 Jun 27;12(4):345–59. doi: 10.1002/qub2.67 (PMC11446534; doi:10.1002/qub2.67)
Supplement: Supplementary file 1 — Table S1 [file QUB2-12-345-s001.pdf]

Supplementary Table S1: List of studies discussed in this review.

Areas	Manuscript Title	Preprint	Journal/Conference	Date of online release	Github	Category
Bioinformatics programming	On the Potential of Artificial Intelligence Chatbots for Data Exploration of Federated Bioinformatics Knowledge Graphs	Arxiv	SeWebMeDa'23: 6th Workshop on Semantic Web solutions for large-scale biomedical data analytics	April 20, 2023	NA	Evaluation
Bioinformatics programming	AI chatbots can boost scientific coding	NA	Nature Ecology & Evolution	April 26, 2023	NA	Evaluation
Bioinformatics programming	Code interpreter for bioinformatics: are we there yet?	NA	Annals of Biomedical Engineering	July 23, 2023	NA	Evaluation
Bioinformatics programming	Biocoder: A benchmark for bioinformatics code generation with contextual pragmatic knowledge	Arxiv	Bioinformatics (in press)	August 31, 2023	https://github.com/gersteinlab/biocoder	Evaluation
Bioinformatics programming	SPARQL Generation: an analysis on fine-tuning OpenLLaMA for Question Answering over a Life Science Knowledge Graph	Arxiv	SWAT4HCLS 2024: The 15th International Conference on Semantic Web Applications and Tools for Health Care and	February 7, 2024	https://github.com/RIKEN-DKO/Generation_SPARQL	Evaluation
Biomedical image understanding	Scientific figures interpreted by chatgpt: Strengths in plot recognition and limits in color perception	BioRxiv	npj Precision Oncology	October 17, 2023	NA	Evaluation
Biomedical image understanding	A pilot study on the efficacy of GPT-4 in providing orthopedic treatment recommendations from MRI reports	NA	Scientific Reports	November 17, 2023	NA	Evaluation
Biomedical text mining	Evaluation of ChatGPT Family of Models for Biomedical Reasoning and Classification	Arxiv	JAMIA	April 5, 2023	https://github.com/shan23chen/HealthLLM_Eval	Evaluation
Biomedical text mining	An extensive benchmark study on biomedical text generation and mining with chatgpt.	BioRxiv	Bioinformatics	April 20, 2023	NA	Evaluation
Biomedical text mining	Opportunities and Challenges for ChatGPT and Large Language Models in Biomedicine and Health	Arxiv	Briefings in Bioinformatics	June 15, 2023	NA	Evaluation
Biomedical text mining	Is ChatGPT a Biomedical Expert?--Exploring the Zero-Shot Performance of Current GPT Models in Biomedical Tasks	Arxiv	11th BioASQ Workshop at CLEF 2023	June 28, 2023	https://github.com/SamyAteia/bioasq	Evaluation
Biomedical text mining	A comprehensive evaluation of large language models on benchmark biomedical text processing tasks	Arxiv	Computers in Biology and Medicine journal	October 6, 2023	https://github.com/tahmedge/llm-eval-biomed	Evaluation
Biomedical text mining	Iterative Prompt Refinement for Mining Gene Relationships from ChatGPT	BioRxiv	International Journal of Artificial Intelligence and Robotics Research (in press)	December 23, 2023	NA	Evaluation
Biomedical text mining	EVLncRNAs 3.0: an updated comprehensive database for manually curated functional long non-coding RNAs validated by low-throughput experiments	NA	Nucleic Acids Research	January 1, 2024	https://www.sdklab-biophysics-dzu.net/EVLncRNAs3/#/	Application
Biomedical text mining	Computational screening of biomarkers and potential drugs for arthrofibrosis based on combination of sequencing and large nature language model	NA	Journal of Orthopaedic Translation	January 20, 2024	https://chenxi2023.shinyapps.io/afdbv1/	Application
Biomedical text mining	A Comprehensive Evaluation of Large Language Models in Mining Gene Interactions and Pathway Knowledge	BioRxiv	Quantitative Biology (in press)	January 24, 2024	https://github.com/Muh-aza/LLM	Evaluation
Chatbots in bioinformatics education	Evaluating a large language model's ability to solve programming exercises from an introductory bioinformatics course	aRxiv	PLoS Computational Biology	March 7, 2023	NA	Evaluation
Chatbots in bioinformatics education	Empowering Beginners in Bioinformatics with ChatGPT	BioRxiv	Quantitative Biology	March 8, 2023	NA	Evaluation
Drug discovery	The capability of ChatGPT in predicting and explaining common drug-drug interactions	NA	Cureus	March 17, 2023	NA	Evaluation
Drug discovery	ChatGPT-powered Conversational Drug Editing Using Retrieval and Domain Feedback	Arxiv	ICLR 2024	May 18, 2023	https://github.com/chao1224/ChatDrug	Application
Drug discovery	What can large language models do in chemistry? a comprehensive benchmark on eight tasks	Arxiv	Advances in Neural Information Processing Systems	May 27, 2023	https://github.com/ChemFoundationModels/ChemLLMBench	Evaluation
Drug discovery	Empowering molecule discovery for molecule-caption translation with large language models: A chatgpt perspective	Arxiv	IEEE TRANSACTIONS ON KNOWLEDGEANDDATAENGINEERING (in press)	June 11, 2023	https://github.com/phenixace/MolReGPT	Evaluation
Drug discovery	Mol-instructions: A large-scale biomolecular instruction dataset for large language models	Arxiv	ICLR 2024	June 13, 2023	https://github.com/zjunlp/Mol-Instructions	Evaluation
Drug discovery	Performance of ChatGPT on the pharmacist licensing examination in Taiwan	NA	Journal of the Chinese Medical Association	July 5, 2023	NA	Evaluation
Drug discovery	Performance of ChatGPT on Chinese national medical licensing examinations: a five-year examination evaluation study for physicians, pharmacists and nurses	medRxiv	BMC Medical Education	August 02, 2023	NA	Evaluation
Drug discovery	Evaluating the performance of ChatGPT in clinical pharmacy: a comparative study of ChatGPT and clinical pharmacists	NA	British journal of clinical pharmacology	August 25, 2023	NA	Evaluation
Drug discovery	Evaluating the sensitivity, specificity, and Accuracy of ChatGPT-3.5, ChatGPT-4, Bing AI, and bard against conventional drug-drug interactions clinical tools	NA	Drug, Healthcare and Patient Safety	September 20, 2023	NA	Evaluation
Drug discovery	Leveraging large language models for predictive chemistry	ChemRxiv	Nature Machine Intelligence	October 17, 2023	https://github.com/kjappelbaum/gptchem	Evaluation
Drug discovery	Examining the Potential of ChatGPT on Biomedical Information Retrieval: Fact-Checking Drug-Disease Associations	NA	Annals of Biomedical Engineering	October 19, 2023	NA	Evaluation
Drug discovery	A generative drug–drug interaction triplets extraction framework based on large language models	NA	Proceedings of the Association for Information Science and Technology	October 22, 2023		Evaluation
Drug discovery	The potential of GPT-4 as a support tool for pharmacists: analytical study using the Japanese national examination for pharmacists	NA	JMIR Medical Education	October 30, 2023	NA	Evaluation
Drug discovery	Fine-tuning large language models for chemical text mining	ChemRxiv	Chemical Science (in press)	November 16, 2023	https://github.com/zw-SIMM/SFTChatGPT_for_chemtext_mining	Evaluation
Drug discovery	ChatGPT in Drug Discovery: A Case Study on Anticocaine Addiction Drug Development with Chatbots	NA	Journal of Chemical Information and Modeling	November 13, 2023	https://github.com/wangru25/SGNC	Application
Drug discovery	Comprehensive evaluation of molecule property prediction with ChatGPT	NA	Methods	January 17, 2024	NA	Evaluation

Genetics	Analysis of large-language model versus human performance for genetics questions	medRxiv	European Journal of Human Genetics	January 28, 2023	NA	Evaluation
Genetics	Can chatgpt understand genetics?	NA	European Journal of Human Genetics	July 5, 2023	NA	Evaluation
Genetics	Is artificial intelligence getting too much credit in medical genetics?	NA	American Journal of Medical Genetics	August 22, 2023	NA	Evaluation
Genetics	Genetic counselors' utilization of ChatGPT in professional practice: A cross-sectional study	NA	American Journal of Medical Genetics	December 8, 2023	NA	Evaluation
Omics	Assessing GPT-4 for cell type annotation in single-cell RNA-seq analysis	BioRxiv	Nature Methods	April 21, 2023	https://github.com/Winnie09/GPTCelltype_Paper	Evaluation
Omics	Genegpt: Augmenting large language models	Arxiv	Bioinformatics	May 16, 2023	https://github.com/ncbi/GeneGPT.	Application
Bioinformatics programming	GenSpectrum Chat: Data Exploration in Public Health Using Large Language Models	Arxiv	NA	May 23, 2023	https://cov-spectrum.org/chat	Application
Bioinformatics programming	BioMANIA: Simplifying bioinformatics data analysis through conversation	BioRxiv	NA	November 1, 2023	https://github.com/batmen-lab/BioMANIA	Application
Bioinformatics programming	Leveraging large language models for data analysis automation	BioRxiv	NA	December 21, 2023	https://github.com/BIMSBbioinfo/mergen-manuscript	Application
Bioinformatics programming	An AI Agent for Fully Automated Multi-omic Analyses	BioRxiv	NA	January 5, 2024	https://github.com/JoshuaChou2018/AutoBA	Application
Bioinformatics programming	BioLLMBench: A Comprehensive Benchmarking of Large Language Models in Bioinformatics	BioRxiv	NA	January 16, 2024	NA	Evaluation
Biomedical image understanding	Accuracy of a vision-language model on challenging medical cases	Arxiv	NA	November 9, 2023	NA	Evaluation
Biomedical image understanding	Performance of multimodal gpt-4v on usmle with image: Potential for imaging diagnostic support with explanations	medRxiv	NA	November 15, 2023	NA	Evaluation
Biomedical image understanding	GPT-4V exhibits human-like performance in biomedical image classification	BioRxiv	NA	January 1, 2024	https://github.com/Winnie09/gptimage	Evaluation
Biomedical image understanding	Hidden Flaws Behind Expert-Level Accuracy of GPT-4 Vision in Medicine	Arxiv	NA	January 16, 2024	NA	Evaluation
Biomedical text mining	Evaluation of GPT and BERT-based models on identifying protein-protein interactions in biomedical text	Arxiv	NA	March 30, 2023	NA	Evaluation
Biomedical text mining	Large language models in biomedical natural language processing: benchmarks, baselines, and recommendations	Arxiv	NA	May 10, 2023	https://github.com/qingyu-qc/gpt_bionlp_benchmark	Evaluation
Biomedical text mining	PlantConnectome: knowledge networks encompassing> 100,000 plant article abstracts	BioRxiv	NA	July 15, 2023	https://connectome.plant.tools/	Application
Biomedical text mining	From answers to insights: Unveiling the strengths and limitations of chatgpt and biomedical knowledge graphs	Res Sq	NA	August 1, 2023	NA	Evaluation
Biomedical text mining	ChatGPT usage in the Reactome curation process	BioRxiv	NA	November 8, 2023	NA	Application
Biomedical text mining	reguloGPT: Harnessing GPT for Knowledge Graph Construction of Molecular Regulatory Pathways	BioRxiv	NA	January 30, 2024	https://github.com/Huang-AI4Medicine-Lab/reguloGPT	Application
Drug discovery	Bayesian optimization of catalysts with in-context learning	Arxiv	NA	April 11, 2023	https://github.com/ur-whitelab/BO-LIFT	Evaluation
Drug discovery	DrugChat: towards enabling ChatGPT-like capabilities on drug molecule graphs	Arxiv	NA	May 18, 2023	https://github.com/UCSD-AI4H/drugchat	Application
Drug discovery	Interactive molecular discovery with natural language	Arxiv	NA	June 21, 2023	https://github.com/Ellenzzn/ChatMol/tree/main	Application
Drug discovery	Instructmol: Multi-modal integration for building a versatile and reliable molecular assistant in drug discovery	Arxiv	NA	November 27, 2023	https://idea-xl.github.io/InstructMol/	Application
Drug discovery	Drugassist: A large language model for molecule optimization	Arxiv	NA	December 28, 2023	https://github.com/blazerye/DrugAssist	Application
Drug discovery	ChemDFM: Dialogue Foundation Model for Chemistry	Arxiv	NA	January 26, 2024	NA	Application
Omics	GeneTuring tests GPT models in genomics	BioRxiv	NA	March 13, 2023	NA	Evaluation

For preprints not yet formally published, those cited to support shared findings across independent works are underlined.
