# Supplementary material for: Bioinformatics and biomedical informatics with ChatGPT: Year one review
Source: Quant Biol. 2024 Jun 27;12(4):345–59. doi: 10.1002/qub2.67 (PMC11446534; doi:10.1002/qub2.67)
Supplement: Supplementary file 2 — Table S2 [file QUB2-12-345-s002.pdf]

Supplementary Table S2: Performance comparison of ChatGPT to baseline models on biomedical text mining tasks.

				ChatGPT								BERT						Alternative LLMs to ChatGPT																			Others
Reference Title	Tasks	Benchmark	Evaluation Metrics	GPT-3.5	3.5 (one-shot)	3.5 (few-shot)	3.5 (few-shot	GPT-4	GPT-4 (one-shot)	GPT-4 (few-shot)	(few-shot CoT)	PubMedBERT	BioLinkBERT-Base	kBERT T-Large	BioBERT	PubmedBert	SciBERT	Claude-2	Claude-Instant	Cohere	Claude-Pro	PaLM-2	Bard	Codellama-34	Wizardlm-70b	Wizardlm-13b	Falcon-180b	Mistral-7b	Chatglm2-6b	Vicuna-7b	Vicuna-33b	Vicuna-13b	llama-2-70b	LLaMa-2-13b	llama-2-7b	Qwen-14b	BoW
An extensive benchmark study on biomedical text generation and mining with chatgpt.	Named entity recognition	BC5-chem	F1 entity-level	60.3	-	-	-	-	-	-	-	93.33	93.75	94.04	-	-	-	-	-	-	-	-	-	-	-	-	-	-	-	-	-	-	-	-	-	-	
	Named entity recognition	BC5-disease	F1 entity-level	51.77	-	-	-	-	-	-	-	85.62	86.1	86.39	-	-	-	-	-	-	-	-	-	-	-	-	-	-	-	-	-	-	-	-	-	-	
	Named entity recognition	NCBI-disease	F1 entity-level	50.49	-	-	-	-	-	-	-	87.82	88.18	88.76	-	-	-	-	-	-	-	-	-	-	-	-	-	-	-	-	-	-	-	-	-	-	
	Named entity recognition	BC2GM	F1 entity-level	37.54	-	-	-	-	-	-	-	84.52	84.9	85.18	-	-	-	-	-	-	-	-	-	-	-	-	-	-	-	-	-	-	-	-	-	-	
	Named entity recognition	JNLPBA	F1 entity-level	41.25	-	-	-	-	-	-	-	80.06	79.03	80.06	-	-	-	-	-	-	-	-	-	-	-	-	-	-	-	-	-	-	-	-	-	-	
	PICO extraction	EBM PICO	Macro F1 word-level	55.59	-	-	-	-	-	-	-	73.38	73.97	74.19	-	-	-	-	-	-	-	-	-	-	-	-	-	-	-	-	-	-	-	-	-	-	
	Relation extraction	ChemProt	Micro F1	34.16	48.64	-	-	-		-	-	77.24	77.57	79.98	-	-	-	-	-	-	-	-	-	-	-	-	-	-	-	-	-	-	-	-	-	-	
	Relation extraction	DDI	Micro F1	51.62	-	-	-	-	-	-	-	82.36	82.72	83.35	-	-	-	-	-	-	-	-	-	-	-	-	-	-	-	-	-	-	-	-	-	-	
	Relation extraction	GAD	Micro F1	52.43	-	-	-	-	-	-	-	82.34	84.39	84.9	-	-	-	-	-	-	-	-	-	-	-	-	-	-	-	-	-	-	-	-	-	-	
	Sentence similarity	BIOSSES	Pearson	43.75	-	-	-	-	-	-	-	92.3	93.25	93.63	-	-	-	-	-	-	-	-	-	-	-	-	-	-	-	-	-	-	-	-	-	-	
	Document classification	HoC	Average Micro F1	51.22	-	-	-	-	-	-	-	82.34	84.39	84.9	-	-	-	-	-	-	-	-	-	-	-	-	-	-	-	-	-	-	-	-	-	-	
	Question answering	PubMedQA	Accuracy	76.45	-	-	-	-	-	-	-	55.84	70.2	72.18	-	-	-	-	-	-	-	-	-	-	-	-	-	-	-	-	-	-	-	-	-	-	
	Question answering	BioASQ	Accuracy	88.57	-	-	-	-	-	-	-	87.56	91.43	94.82	-	-	-	-	-	-	-	-	-	-	-	-	-	-	-	-	-	-	-	-	-	-	
Large language models in biomedical natural language processing: benchmarks, baselines, and recommendations	Named entity recognition	BC5CDR-chemical	F1 entity-level	68.36	72.1	-	-	81.9	82.43	-	-	93.5	-	-	-	-	-	-	-	-	-	-	-	-	-	-	-	-	-	-	-	-	-	-	-	-	
	Named entity recognition	NCBI-disease	F1 entity-level	38.02	42.74	-	-	57.85	58.39	-	-	89.86	-	-	-	-	-	-	-	-	-	-	-	-	-	-	-	-	-	-	-	-	-	-	-	-	
	Relation extraction	ChemProt	Micro F1	57.43	57.71	-	-	66.18	66.82	-	-	78.32	-	-	-	-	-	-	-	-	-	-	-	-	-	-	-	-	-	-	-	-	-	-	-	-	
	Relation extraction	DDI2013	Micro F1	33.49	34.34	-	-	63.25	61.76	-	-	80.23	-	-	-	-	-	-	-	-	-	-	-	-	-	-	-	-	-	-	-	-	-	-	-	-	
	Multi-label classification	HoC	Label-wise macro F1	65.72	69.32	-	-	74.74	74.02	-	-	89.15	-	-	-	-	-	-	-	-	-	-	-	-	-	-	-	-	-	-	-	-	-	-	-	-	
	Multi-label classification	LitCovid	Label-wise macro F1	63.9	65.31	-	-	67.46	68.39	-	-	87.24	-	-	-	-	-	-	-	-	-	-	-	-	-	-	-	-	-	-	-	-	-	-	-	-	
	Semantic similarity and reasoning	PubMedQA	Pearson	35.53	30.11	-	-	43.74	53.61	-	-	36.76	-	-	-	-	-	-	-	-	-	-	-	-	-	-	-	-	-	-	-	-	-	-	-	-	
	Semantic similarity and reasoning	BIOSSES	Pearson	87.86	91.94	-	-	88.32	89.22	-	-	93.32	-	-	-	-	-	-	-	-	-	-	-	-	-	-	-	-	-	-	-	-	-	-	-	-	
	Text summarization	summarization	ROUGE-1	6.08	23.2	-	-	39.97	40.54	-	-	44.89	-	-	-	-	-	-	-	-	-	-	-	-	-	-	-	-	-	-	-	-	-	-	-	-	
	Text summarization	MS^2	ROUGE-1	17.31	12.11	-	-	18.77	19.19	-	-	20.79	-	-	-	-	-	-	-	-	-	-	-	-	-	-	-	-	-	-	-	-	-	-	-	-	
	Text simplification	Cochrane PLS	Flesch-Kincaid score	13.0505	13.18	-	-	12.0001	13.12	-	-	12.64	-	-	-	-	-	-	-	-	-	-	-	-	-	-	-	-	-	-	-	-	-	-	-	-	
	Text simplification	PLOS text simplification	Flesch-Kincaid score	14.0605	13.92	-	-	13.219	13.24	-	-	14.66	-	-	-	-	-	-	-	-	-	-	-	-	-	-	-	-	-	-	-	-	-	-	-	-	
Evaluation of ChatGPT Family of Models for Biomedical Reasoning and Classification	Classification	"Advice in discussion sections"	Micro F1	50.6	51.3	47.5	67.1	50.9	-	-	64.8	-	-	-	80 (100% fine-tuning)	-	-	-	-	-	-	-	-	-	-	-	-	-	-	-	-	-	-	-	-	-	59.3 (100% fine-tuning)
	Classification	"Advice in unstructured abstracts"	Micro F1	48.9	55.4	40.3	67	47.8	-	-	71.2	-	-	-	82.1 (100% fine-tuning)	-	-	-	-	-	-	-	-	-	-	-	-	-	-	-	-	-	-	-	-	-	64 (100% fine-tuning)
	Classification	"Advice in structured abstracts"	Micro F1	54.8	59.3	49.5	71.8	51.7	-	-	77	-	-	-	90.2 (100% fine-tuning)	-	-	-	-	-	-	-	-	-	-	-	-	-	-	-	-	-	-	-	-	-	75.3 (100% fine-tuning)
	Reasoning	"Causal relation detection"	Micro F1	28.8	39.6	54.2	64.9	30.1	-	-	68.2	-	-	-	85.1 (100% fine-tuning)	-	-	-	-	-	-	-	-	-	-	-	-	-	-	-	-	-	-	-	-	-	67.5 (100% fine-tuning)
A comprehensive evaluation of large language models on benchmark biomedical text processing tasks	Relation extraction	BC5CDR	F1	43.29	-	-	-	-	-	-	-	-	-	-	-	-	-	53.37	-	-	-	54.3	-	-	-	-	-	-	-	-	-	-	-	53.28	-	-	-
	Relation extraction	KD-DTI	F1	29.74	-	-	-	-	-	-	-	-	-	-	-	-	-	28.84	-	-	-	38.44	-	-	-	-	-	-	-	-	-	-	-	24.21	-	-	-
	Relation extraction	DDI	F1	46.43	-	-	-	-	-	-	-	-	-	-	-	-	-	42.62	-	-	-	22.5	-	-	-	-	-	-	-	-	-	-	-	24.03	-	-	-
	Text classification	HoC	F1	59.26	-	-	-	-	-	-	-	-	-	-	-	-	-	34.93	-	-	-	61.03	-	-	-	-	-	-	-	-	-	-	-	41.82	-	-	-
	Text classification	LitCovid	F1	29.63	-	-	-	-	-	-	-	-	-	-	-	-	-	7.6	-	-	-	37.5	-	-	-	-	-	-	-	-	-	-	-	11.34	-	-	-
	Question answering	PubMedQA	Accuracy	54.4	-	-	-	-	-	-	-	-	-	-	-	-	-	57.2	-	-	-	59.6	-	-	-	-	-	-	-	-	-	-	-	61.4	-	-	-
	Question answering	MediQA-2019	Accuracy	73.26	-	-	-	-	-	-	-	-	-	-	-	-	-	65.13	-	-	-	52.12	-	-	-	-	-	-	-	-	-	-	-	56.01	-	-	-
	Entity linking dataset	BC5CDR	Recall@1	54.9	-	-	-	-	-	-	-	-	-	-	-	-	-	78.01	-	-	-	52.14	-	-	-	-	-	-	-	-	-	-	-	66.52	-	-	-
	Entity linking dataset	Cometa	Recall@1	43.45	-	-	-	-	-	-	-	-	-	-	-	-	-	53.29	-	-	-	48.76	-	-	-	-	-	-	-	-	-	-	-	40.67	-	-	-
	Entity linking dataset	NCBI	Recall@1	52.19	-	-	-	-	-	-	-	-	-	-	-	-	-	70.21	-	-	-	38.44	-	-	-	-	-	-	-	-	-	-	-	59.17	-	-	-
	Named entity recognition	BC2GM	F1	31.99	-	-	-	-	-	-	-	-	-	-	-	-	-	40.45	-	-	-	32.75	-	-	-	-	-	-	-	-	-	-	-	5.95	-	-	-
	Named entity recognition	BC4CHEMD	F1	26.01	-	-	-	-	-	-	-	-	-	-	-	-	-	35.18	-	-	-	25.83	-	-	-	-	-	-	-	-	-	-	-	6.64	-	-	-
	Named entity recognition	BC5CDR-chem	F1	41.25	-	-	-	-	-	-	-	-	-	-	-	-	-	58.05	-	-	-	48.08	-	-	-	-	-	-	-	-	-	-	-	12.21	-	-	-
	Named entity recognition	BC5CDR-disease	F1	32.26	-	-	-	-	-	-	-	-	-	-	-	-	-	50.13	-	-	-	33.72	-	-	-	-	-	-	-	-	-	-	-	5.68	-	-	-
	Named entity recognition	JNLPBA	F1	31.89	-	-	-	-	-	-	-	-	-	-	-	-	-	34.62	-	-	-	21.18	-	-	-	-	-	-	-	-	-	-	-	4.3	-	-	-
	Named entity recognition	NCBI-disease	F1	33.39	-	-	-	-	-	-	-	-	-	-	-	-	-	45.75	-	-	-	31.15	-	-	-	-	-	-	-	-	-	-	-	4.58	-	-	-
	Named entity recognition	linnaeus	F1	5.14	-	-	-	-	-	-	-	-	-	-	-	-	-	13.91	-	-	-	6.44	-	-	-	-	-	-	-	-	-	-	-	1.42	-	-	-
	Named entity recognition	s800	F1	15.57	-	-	-	-	-	-	-	-	-	-	-	-	-	24.07	-	-	-	16.96	-	-	-	-	-	-	-	-	-	-	-	1.87	-	-	-
	Text summarization	iCliniq	ROUGE-1	30.5	-	-	-	-	-	-	-	-	-	-	-	-	-	28.8	-	-	-	21.9	-	-	-	-	-	-	-	-	-	-	-	20	-	-	-
	Text summarization	HealthCareMagic	ROUGE-1	28.1	-	-	-	-	-	-	-	-	-	-	-	-	-	24.4	-	-	-	25.9	-	-	-	-	-	-	-	-	-	-	-	16.7	-	-	-
	Text summarization	MeQSum	ROUGE-1	30	-	-	-	-	-	-	-	-	-	-	-	-	-	31.7	-	-	-	31.5	-	-	-	-	-	-	-	-	-	-	-	21.2	-	-	-
	Text summarization	MEDIQA-QS	ROUGE-1	30.6	-	-	-	-	-	-	-	-	-	-	-	-	-	32	-	-	-	29.7	-	-	-	-	-	-	-	-	-	-	-	23.3	-	-	-
	Text summarization	MEDIQA-MAS	ROUGE-1	38.9	-	-	-	-	-	-	-	-	-	-	-	-	-	13.4	-	-	-	15.3	-	-	-	-	-	-	-	-	-	-	-	13.7	-	-	-
	Text summarization	MEDIQA-ANS	ROUGE-1	28.7	-	-	-	-	-	-	-	-	-	-	-	-	-	28.6	-	-	-	25.4	-	-	-	-	-	-	-	-	-	-	-	28	-	-	-
	Text summarization	PLOS (Abstract)	ROUGE-1	39.65	-	-	-	-	-	-	-	-	-	-	-	-	-	42.25	-	-	-	25.09	-	-	-	-	-	-	-	-	-	-	-	41.78	-	-	-
	Text summarization	Summarization)	ROUGE-1	39.13	-	-	-	-	-	-	-	-	-	-	-	-	-	36.16	-	-	-	30.7	-	-	-	-	-	-	-	-	-	-	-	36.33	-	-	-
Evaluation of GPT and BERT-based models on identifying protein-protein interactions in biomedical text	Relation extraction	LLL	F1-Score	76.72 (with protein dictionary)	-	-	-	86.49 (with protein dictionary)	-	-	-	-	-	-	86.84	85.42	84.66	-	-	-	-	-	-	-	-	-	-	-	-	-	-	-	-	-	-	-	-
	Relation extraction	IEPA	F1-Score	31.25 (with protein dictionary)	-	-	-	48.37 (with protein dictionary)	-	-	-	-	-	-	78.81	78.49	75.53	-	-	-	-	-	-	-	-	-	-	-	-	-	-	-	-	-	-	-	-
	Relation extraction	HPRD50	F1-Score	52.9 (with protein dictionary)	-	-	-	57.24 (with protein dictionary)	-	-	-	-	-	-	74.95	79.65	74.67	-	-	-	-	-	-	-	-	-	-	-	-	-	-	-	-	-	-	-	-
A Comprehensive Evaluation of Large Language Models in Mining Gene Interactions and Pathway Knowledge	Relation extraction	Gene regulatory relations	F1	0.1485	-	-	-	0.4448	-	-	-	-	-	-	-	-	-	0.422	0.326	0.374	0.439	0.261	0.279	0.144	0.045	0.096	0.279	0.123	0.182	0.123	0.141	0.181	0.158	0.091	0.192	0.101	-
	Relation extraction	KEGG pathway recognition	Jaccard similarity	0.1358	-	-	-	0.2778	-	-	-	-	-	-	-	-	-	0.189	0.152	0.209	0.266	0.105	0.192	0.162	0.135	0.146	0.224	0.143	0.088	0.174	0.166	0.154	0.154	0.171	0.221	0.084	-

Values are extracted from corresponding reference listed in the first column. In red are numbers that are no better than at least one kind of GPT.
