# Supplementary material for: Bioinformatics and biomedical informatics with ChatGPT: Year one review
Source: Quant Biol. 2024 Jun 27;12(4):345–59. doi: 10.1002/qub2.67 (PMC11446534; doi:10.1002/qub2.67)
Supplement: Supplementary file 3 — Table S3 [file QUB2-12-345-s003.pdf]

Supplementary Table S3: Performance comparison of ChatGPT to baseline models on drug discovery tasks.

Reference Title	Tasks	Benchmark	Evaluation Metrics	ChatGPT			Baseline models																		
				GPT-3.5	GPT-3.5 (fine-tuned)	GPT-4	Davinci-003	GAL-30B	MolT5-Large	LLama-2-7B	Llama2-13B-chat	BART-base	T5-base	T5-large	MolT5-base	MolT5-large	text-ada-001 (fine-tuned)	Ridge Regression	Neighbor (KNN)	ada embeddings	SolTranNet	SMILES-BERT	MolBERT	Regression Transformer	MolFormer
What can large language models do in chemistry? a comprehensive benchmark on eight tasks	Name Prediction ("smiles2formula")	PubChem	Accuracy	0.052 ('Scaffold,k=20')	-	0.086 ('Scaffold,k=20')	0.006 ("Scaffold,k=20")	-	-	-	0.01 ("Scaffold,k=20")	-	-	-	-	-	-	-	-	-	-	-	-	-	-
	Property Prediction	BBBP	F1	0.463 ('Scaffold,k=20')	-	0.587 ('Scaffold,k=20')	0.378 ('Scaffold,k=20')	0.074 ('Scaffold,k=20')	-	-	0.002 ('Scaffold,k=20')	-	-	-	-	-	-	-	-	-	-	-	-	-	-
	Property Prediction	HIV	F1	0.406 ('Scaffold,k=20')	-	0.666 ('Scaffold,k=20')	0.649 ('Scaffold,k=20')	0.025 ('Scaffold,k=20')	-	-	0.045 ('Scaffold,k=20')	-	-	-	-	-	-	-	-	-	-	-	-	-	-
	Property Prediction	BACE	F1	0.807 ('Scaffold,k=20')	-	0.797 ('Scaffold,k=20')	0.832 ('Scaffold,k=20')	0.014 ('Scaffold,k=20')	-	-	0.069 ('Scaffold,k=20')	-	-	-	-	-	-	-	-	-	-	-	-	-	-
	Property Prediction	Tox21	F1	0.529 ('Scaffold,k=20')	-	0.563 ('Scaffold,k=20')	0.518 ('Scaffold,k=20')	0.077 ('Scaffold,k=20')	-	-	0.047 ('Scaffold,k=20')	-	-	-	-	-	-	-	-	-	-	-	-	-	-
	Property Prediction	ClinTox	F1	0.369 ('Scaffold,k=20')	-	0.736 ('Scaffold,k=20')	0.85 ('Scaffold,k=20')	0.081 ('Scaffold,k=20')	-	-	0.001 ('Scaffold,k=20')	-	-	-	-	-	-	-	-	-	-	-	-	-	-
	Yield Prediction	Buchwald-Hartwig	Accuracy	0.585 ('random, k = 8')	-	0.8 ('random, k = 8')	0.467 ('random, k = 8')	0	-	-	0.008	-	-	-	-	-	-	-	-	-	-	-	-	-	-
	Yield Prediction	Suzuki-Miyaura	Accuracy	0.542 ('random, k = 8')	-	0.764 ('random, k = 8')	0.341 ('random, k = 8')	0.008	-	-	0.006	-	-	-	-	-	-	-	-	-	-	-	-	-	-
	Reaction Prediction	USPTO-Mixed	Accuracy	0.184 ('Scaffold, k=20')	-	0.23 ('Scaffold, k=20')	0.218 ('Scaffold, k=20')	0.036 ('Scaffold, k=5')	-	-	0.032 ('Scaffold, k=20')	-	-	-	-	-	-	-	-	-	-	-	-	-	-
	Reagents Selection	Suzuki-Miyaura	Top-1 Accuracy	0.4	-	0.299	0.178	0.107	-	-	0.145	-	-	-	-	-	-	-	-	-	-	-	-	-	-
	Retrosynthesis	USPTO-50k	Top-1Accuracy	0.022 ('Scaffold, k=20')	-	0.096 ('Scaffold, k=20')	0.122 ('Scaffold, k=20')	0.016 ('Scaffold, k=5')	-	-	0 ('Scaffold, k=20')	-	-	-	-	-	-	-	-	-	-	-	-	-	-
	Text-Based Molecule Design	ChEBI-20	BLEU	0.479 ('Scaffold,k=10')	-	0.816 ('Scaffold, k=10')	0.741 ('Scaffold, k=10')	0.004	0.601	-	0.626 ('Scaffold, k=10')	-	-	-	-	-	-	-	-	-	-	-	-	-	-
	Molecule Captioning	ChEBI-20	BLEU-2	0.468 ('Scaffold,k=10')	-	0.464 ('Scaffold,k=10')	0.488 ('Scaffold,k=10')	0.008	0.482	-	0.197 ('Scaffold,k=10')	-	-	-	-	-	-	-	-	-	-	-	-	-	-
Empowering molecule discovery for molecule-caption translation with large language models: A chatgpt perspective	Molecule Captioning	ChEBI-20	BLEU-2	0.565 ('10-shot MolReGPT')	-	0.607 ('10-shot MolReGPT')	-	-	-	0.489 (2-shot MolReGPT)	-	-	0.511	0.558	0.54	0.594	-	-	-	-	-	-	-	-	-
	Text-Based Molecule Design	ChEBI-20	BLEU	0.79 ('10-shot MolReGPT')	-	0.857 ('10-shot MolReGPT')	-	-	-	0.693 (2-shot MolReGPT)	-	-	0.762	0.854	0.769	0.854	-	-	-	-	-	-	-	-	-
Bayesian optimization of catalysts with in-context learning	Property Prediction ("aqueous solubility")	ESOL	RMSE (the lower the better)	-	-	0.773 ('topk')	1.185 ('topk')	-	-	-	-	-	-	-	-	-	1.558 ('topk')	-	2.443	2.652 ('topk')	2.99	0.47	0.531	0.73	0.278
	Property Prediction ("reaction yield")	Nguyen et al. (2020) ACS Catal 10(2):921-932	RMSE (the lower the better)	-	-	2.683 ('topk')	2.652 ('topk')	-	-	-	-		-	-	-	-	1.936 ('topk')	2.114 ('topk')	3.247 ('topk')	4.173 ('topk')	-	-	-	-	-
Fine-tuning Large Language Models for Chemical Text Mining	Action Sequence extraction	Specified in Suppl Table but not accessible at time of writing	BLEU	49.5 ('30-shots')	84.8 (fine-tuned with 1060 samples)	65.0 ('60-shots')	-	-	-	-	81.6(fine-tuned with 1060 samples)	74.4(fine-tuned with 1060 samples)	84.1(fine-tuned with 1060 samples)	-	-	-	-	-	-	-	-	-	-	-	-

Values are extracted from corresponding reference listed in the first column. In red are numbers that are no better than at least one kind of GPT. In paratheses are prompting strategies or additional settings detailed in the corresponding litetature.
